# The effect of soy isoflavones on non-alcoholic fatty liver disease and the level of fibroblast growth factor-21 and fetuin A

**DOI:** 10.1038/s41598-024-55747-6

**Published:** 2024-03-01

**Authors:** Asal Neshatbini Tehrani, Behzad Hatami, Bizhan Helli, Zahra Yari, Ghazal Daftari, Amin Salehpour, Mehdi Hedayati, Elmira Khalili, Seyed Ahmad Hosseini, Azita Hekmatdoost

**Affiliations:** 1 Student Research Committee, Ahvaz Jundishapur University of Medical Sciences, Ahvaz, Iran; 2 Department of Nutrition, School of Allied Medical Sciences, Ahvaz Jundishapur University of Medical Sciences, Ahvaz, Iran; 3 Nutrition and Metabolic Diseases Research Center, Clinical Research Institute, Ahvaz Jundishapur University of Medical Sciences, Ahvaz, Iran; 4 Gastroenterology and Liver Diseases Research Center, Research Institute for Gastroenterology and Liver Diseases, Shahid Beheshti University of Medical Sciences, Tehran, Iran; 5 Department of Nutrition Research, National Nutrition and Food Technology Research Institute and Faculty of Nutrition Sciences and Food Technology, Shahid Beheshti University of Medical Sciences, Tehran, Iran; 6 School of Medicine, Tehran University of Medical Sciences, Tehran, Iran; 7 School of Public Health, Occupational Health Research Center, Iran Universityof Medical Sciences, Tehran, Iran; 8 Cellular and Molecular Endocrine Research Center, Research Institute for Endocrine Sciences, Shahid Beheshti University of Medical Sciences, Tehran, Iran; 9 Clinical Nutrition and Dietetics Department, Faculty of Nutrition Sciences and Food Technology, National Nutrition and Food Technology Research Institute, Shahid Beheshti University of Medical Sciences, No 7, West Arghavan St., Farahzadi Blvd., P. O. Box: 19395-4741, Tehran, 1981619573 Iran

**Keywords:** Diseases, Gastroenterology, Medical research

## Abstract

A two-arm randomized open labeled controlled clinical trial was conducted on 50 patients with non-alcoholic fatty liver disease (NAFLD). Subjects were randomized to either receive two tablets of soy isoflavone (100 mg/day) or placebo. At week 12, the serum levels of alanine amino transferase (ALT), aspartate amino transferase (AST) and controlled attenuation parameter (CAP) score were significantly decreased only in the soy isoflavone group (P < 0.05). A significant decline in the gamma glutamyl transferase (GGT) level was observed only in the placebo group (P = 0.017). A significant increase in the serum level of fetuin A was shown in both groups at the end of the trial with a significantly greater increment in the soy isoflavone group compared to the placebo group (P < 0.05). The changes in the serum level of FGF-21 were not significant in any of the two groups. Steatosis grade significantly improved only in the soy isoflavone group (P = 0.045). There was no significant change in the fibrosis grade in the groups. Soy isoflavone intake led to a decrease in ALT, AST, CAP score, steatosis grade and an increase in the level of fetuin A. However, no significant changes were observed in the fibrosis grade and serum levels of GGT and FGF-21.

## Introduction

The burden of Nonalcoholic fatty liver disease (NAFLD) and its associated complications is substantial and growing globally^1^. The pathology is due to fat accumulation in the hepatocytes, which may gradually proceed to fibrosis, cirrhosis or hepatocellular carcinoma^2^. The prevalence of NAFLD depends on age, geographic area, gender and is estimated to be 15–30% in the general population^3^. A recent meta-analysis reported that approximately 35% of Iranian people suffer from NAFLD, which is a considerable rate^4^. Several factors including physical activity, body mass index(BMI), dietary intake and smoking can play a key role in the occurrence of NAFLD^5^. Although, life style and dietary modification are the most important strategies for NAFLD treatment, compliance rate is usually low and pharmacological intervention is generally ineffective^6^. Several studies have demonstrated that some bioactive and functional dietary compounds along with lifestyle interventions provide synergistic effects in the treatment of NAFLD^7–9^. Soy isoflavones are similar to the estrogenic but non-steroidal components found in legumes^10^. Soy products like soybeans are rich in isoflavones, including glycitein, genistein and daidzein, which bind to various sugars to make nalonylglucosides, acetylglucosides and glycosides^11^. The beneficial effects of soy consumption on reducing the progression of NAFLD have been shown in epidemiological studies^12–14^. In addition, higher concentration of isoflavones in soy protein concentrate have been reported to be related to greater protection against NAFLD^15^. The majority of the previous studies have implied the positive role of soy isoflavones on NAFLD through regulating mechanisms on some enzymes such as peroxisome proliferator-activated receptors (PPARs), fatty acid β-oxidation, and oxidative stress^16–19^. Fibroblast growth factor-21 (FGF-21) belongs to FGF superfamily, which are mainly produced by the liver. According to the findings of previous research, FGF-21 level increases in cases of obesity, diabetes and hypertriglyceridemia^20–22^ and also, in patients who have been diagnosed with liver damage according to their biochemical results^23^. Several metabolic functions have been attributed to FGF-21 including the regulation of lipids, glucose, insulin and energy homeostasis^24^. According to an in vivo study, increased levels of FGF-21 indicate a propensity toward the advancement of end-stage NAFLD^25^. Fetuin A is a serum protein which is involved in metabolic syndrome. Impeding the auto phosphorylation of insulin receptors^26^, causing insulin resistance^27^, stimulating the expression of inflammatory cytokines and prohibiting the expression of adiponectin with anti-inflammatory effects^28^ are some of the fetuin A mechanisms that lead to metabolic syndrome. Liver fat accumulation is significantly correlated with the elevated serum level of fetuin A in subjects prone to metabolic syndrome^29,30^. This study was designed to assess the effect of soy isoflavones on NAFLD management and serum levels of FGF-21 and fetuin A.

## Methods and materials

### Enrollment and eligibility criteria

**Figure 1:**
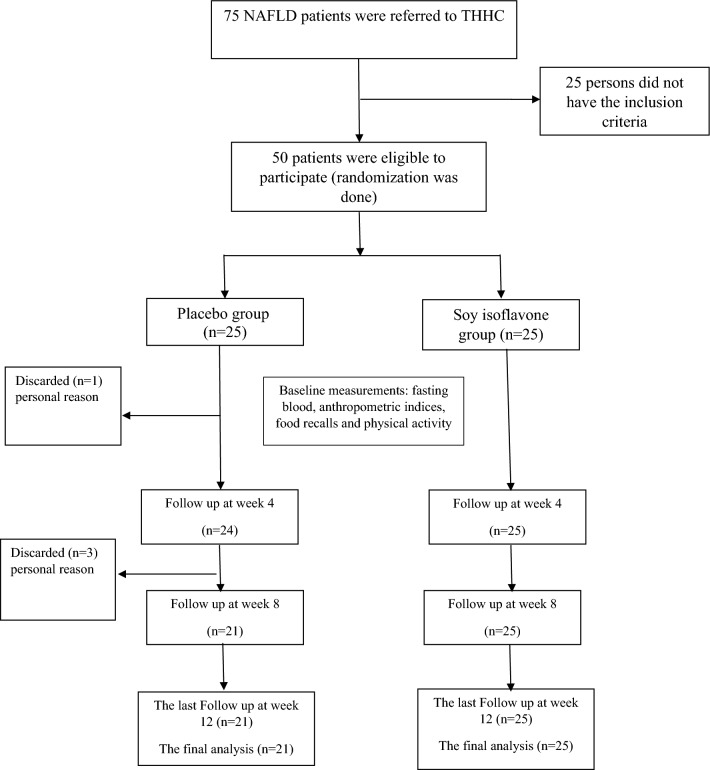
Study flow chart.

In this double blinded, placebo-controlled randomized clinical trial with two parallel groups, 75 ambulatory patients with NAFLD volunteered to participate. Fifty patients were eligible and entered the study but four patients declined to continue the study. Hence, 46 patients (25 and 21 subjects accredited to the soy isoflavone and placebo groups, respectively) completed the study (Fig. 1). Patients with diagnosis of NAFLD and grade ≥ 2 steatosis, confirmed by Fibroscan, were collected from Taleghani Hospital Hepatology Clinic (THHC) in Tehran, Iran. The inclusion criteria were (1) no allergy to soy or massive intake of soy products; (2) 18 years of age or older; (3) not having chronic diseases such as cardiovascular, renal, respiratory, liver, auto immune, malignancy, Cushing syndrome, hepatitis, thyroid dysfunction, biliary disorders, cirrhosis, gastrointestinal disorders with effect on food absorption, diabetes mellitus and psychiatric conditions that interfere with agreement to participate in the study or signing the consent form; (4) alcohol consumption ≥ 10 g/day (safe limit of alcohol intakes are 20 g/day and 30 g/day in women and men with NAFLD, respectively, with one unit equivalent to 10 g intake^31,32^; (5) diagnosis of NAFLD grade two or higher in the Fibroscan test (CAP > 260 dB/m); (6) no history of planned weight loss or bariatric surgery in the last 6 months; (7) not being lactating or pregnant; (8) not smoking; (9) not taking drugs with probable effect on liver such as vitamin E, tamoxifen, lithium, methotrexate, ursodeoxycholic acid, phenytoin, metformin and corticosteroids; (10) not adhering to specific diets such as low caloric diets during the last 6 months.

This information obtained from the participants through a face-to-face clinical interview by a structured questionnaire. The accuracy of information checked by each person’s medical file kept in the hospital. Before the interview, the subjects were ensured that their information would be kept confidentially.

### Study design and follow-up

The minimum sample size calculated for each group was 21 according to the Fibroscan controlled attenuation parameter (CAP) score and detection of a 10 unit difference in the mean CAP score with a power of 80% (β = 20%). To consider the potential loss of subjects, 25 patients in each group were enrolled^33^. The method by which the sample size was calculated is based on the following equation^34^.

$$\begin{aligned} & {\text{n}}_{1} = 10 \\ & {\text{S}}_{1} = 34 \\ & {\text{n}}_{2} = 10 \\ & {\text{S}}_{2} = 23 \\ & {\text{Z}}_{{1 - {\text{ }}\alpha /2}} = 1.96 \\ & {\text{Z}}_{{1 - \beta }} = 0.84 \\ & \mu _{1} - \mu _{2} = 25 \\ & {\text{S}}_{p} ^{2} = \frac{{\left( {{\text{n}}_{{\text{1}}} - {\text{1}}} \right){\text{S}}_{1} ^{2} + {\text{ }}\left( {{\text{n}}_{{\text{2}}} - {\text{1}}} \right){\text{S}}_{2} ^{2} }}{{{\text{n}}_{1} + {\text{ n}}_{2} - {\text{ 2}}}} = 825 \\ & {\text{n}} = \frac{{{\text{2S}}^{2} \left( {{\text{Z}}_{{1 - \alpha /2}} + {\text{ Z}}_{{1 - \beta }} } \right)^{2} }}{{\left( {\mu _{1} - \mu _{2} } \right)^{{\text{2}}} }} = 21 \\ \end{aligned}$$

Patients were blocked randomized and stratified by menopause status and gender, then by a table of randomization number, the participants were assigned to take either soy isoflavones or placebo tablets for 12 weeks. In the soy isoflavone group, patients took 100 mg soy isoflavone in the form of two tablets per day. According to previous studies, prescribing 100 mg/day soy isoflavones has no adverse effects^35–37^. Each soy isoflavone 50 mg tablet contained 1.49 mg of genistein, 31.86 mg of genistin, 1.75 mg of daidzein, 13.21 mg of daidzin, 0.55 mg of glycitein and 1.14 mg of glycitin. The placebo tablets contained starch. The placebo and soy isoflavone tablets were similar in smell, taste, and appearance. Soy isoflavone and placebo tablets were both made by Gol Daru Pharmaceutical Company, Esfahan, Iran. The bottles containing tablets were labeled as A and B by a third party to make the study double-blinded. Thus, researchers and participants were not aware of the content of bottles. Patients have been followed up in weeks 4, 8 and 12 from the study started. Participant’s baseline characteristics were collected in the first visit. Every 4 weeks, the participants were given placebo or soy isoflavone tablets. The compliance rate was assessed by calculating the remaining tablets at each visit. The patients who used less than 90% of tablets were excluded from the study. Life style modification recommendations like physical activity of 30 min for 3 days per week with medium intensity and food intake consults provided by clinical guidelines of NIH and the North American Association for the Study of Obesity were given to both groups^38^. This study is conducted according to CONSORT reporting guidelines^39^.

### Measurements

Anthropometric parameters including weight, hip and waist circumferences were obtained at the beginning and end of the study (weeks 0 and 12, respectively). Height with the bare feet, waist circumference at the narrowest and hip circumference at the widest part were measured using a non-stretch portable meter and were reported to the nearest of 0.5 cm. A scale with an accuracy of 100 g was used for weight measurement. BMI was calculated as weight in kilograms divided to height in squared meters. Waist circumference divided to hip circumference, both in centimeters, was used to report waist to hip ratio (WHR). 3-day dietary recalls (1 weekend and 2 weekdays) were obtained at the starting (week 0) and end of the study (week 12) to assess the dietary intake. A dietitian monitored the dietary data in terms of completeness and Nutritionist 4 software was used to analyze the dietary data (N Squared Computing, San Bruno, CA, USA). Physical activity levels in weeks 0 and 12 were evaluated by a semi-quantitative questionnaire validated in the Iranian population and reported as metabolic equivalent hours per day (MET.h.d). The participants underwent 12-h fasting and 7 ml of blood sample was collected. After remaining at room temperature for 20 min, the centrifugation was performed for 10 min at 2000 rpm. Serum samples were frozen and stored in microtubes at − 70 °C until the start of the analysis. For measuring the serum level of aspartate amino transferase (AST), alanine amino transferase (ALT), and gamma glutamyl transferase (GGT) through enzymatic methods, Delta Darman Part test kits (Delta darman part, Tehran, Iran) were applied. The serum concentrations of FGF-21 and fetuin A were measured via ELISA commercial kits (ZellBio GmbH Veltlinerweg 29, 89075, Ulm, Germany). Fibroscan (Echosense, France) was conducted to assess liver steatosis and fibrosis at study initiation and at the end of week 12. Fibrosis was graded as mild (grade 1), moderate (grade 2), and severe (grade 3), based on Fibroscan patient score (kPa). The cut off points to categorize the fibrosis state are as follow: F1–F2: 2.9–3.5 kPa; F2–F3: > 3.5–4 kPa; F3–F4: > 4–5 kPa; F4 > 5 kPa^40^. Steatosis was graded as normal (grade 0), mild (grade 1), moderate (grade 2) and severe (grade 3) based on CAP score (dB/m). The cut off points to categorize the steatosis state are as follow: grade 1: 271.1 dB/m; grade 2: 303.7 dB/m; grade3: 326.7 dB/m^41^. In order to restrain the biases related to intra-observer, the same technicians and the same equipment were applied. The individuals responsible for the measurements were blinded to all study procedures.

## Primary and secondary outcomes

The primary outcome of the study was a significant reduction in hepatic steatosis score through the Fibroscan measurements. Secondary outcomes were changes in serum concentrations of hepatic enzymes, FGF-21 and fetuin A and changes to anthropometric variables.

### Statistical analysis

We used Statistical Package for the Social Sciences (SPSS, Inc., Chicago, IL, USA, version 21.0) to analyze the data of current study. Kolmogorov–Smirnov test was used to check the normality of the variables. The categorial and quantitative variables were reported in frequency (percentages) and mean ± standard deviation (SD), respectively. For comparing the groups at the week 0 and 12, we used the unpaired t-test. To compare the means before and after supplementation within the groups, the student’s t-test was applied. The analysis of covariance (ANCOVA) was performed to adjust the confounding factors’ effect. To perform ANCOVA, the baseline value of each parameter and the mean changes of WHR, BMI, physical activity level and energy intake were considered. For comparing the categorical variables between and within the two groups, Mann–Whitney U test and Wilcoxon signed-rank test was conducted, respectively. Statistical significance was considered at < 0.05 according to two-sided tests.

### Ethical consideration

The participants were informed about the study and it was mentioned that they are free to quit the study at any time and for any reason. The informed consent form was signed and dated by each participant before the study initiation. The Ethics committee of Ahvaz Jundishapur University of Medical Sciences approved the protocol of the present clinical research (IR.AJUMS.REC.1401.155). The study was in comply with the Declaration of Helsinki. This study’s registration date/number are 25.10.2023/NCT06101433 at ClinicalTrials.gov.

## Results

### Characteristics of participants

As shown in the flow chart (Fig. 1), in the placebo group, four participants dropped out for personal reasons. The degree of compliance for our participants was over 90%. In the present study, no side-effects were observed. The primary variables of subjects attributed to both groups are depicted in Table 1. Except for ALT level, which was significantly higher in the placebo group compared to the soy isoflavone group at study initiation, no significant differences regarding other baseline characteristics were observed between the patients in both groups. The dietary information and physical activity are shown in Table 2. As we can see, in subjects accredited to the soy isoflavone group, a significant decrement was observed in regard to PUFA w-6 and vitamin E intake at the end of the trial in comparison with the baseline (10.57 ± 4.88 vs 8.53 ± 2.54; P = 0.028 and 37.73 ± 16.76 vs 30.68 ± 8.56; P = 0.029, respectively). Comparison of physical activity and other dietary data in both groups and between the two groups did not show any significant statistical difference.

**Table 1:** The characteristics of the subjects at study initiation ^a^.

Parameters	Placebo group (n = 21)	Soy isoflavone group (n = 25)	*P* value ^b^
Gender			0.766
Male	10 (47.60)	10 (40.0)	
Female	11 (52.40)	15 (60.0)	
Age			
Male	46.0 ± 14.10	47.60 ± 14.98	0.809
Female	52.09 ± 5.73	51.93 ± 11.15	0.963
Menopause status (females)			1.00
*Yes*	5 (45.5)	6 (40.0)	
*No*	6 (54.5)	9 (60.0)	
Anthropometric variables			
Height (cm)	168.90 ± 11.0	166.28 ± 9.93	0.770
Weight (kg)	85.42 ± 14.34	84.0 ± 18.09	0.772
WC (cm)	97.19 ± 8.54	98.56 ± 11.99	0.664
HC (cm)	113.43 ± 10.06	112.88 ± 9.74	0.852
BMI (kg/m^2^)	29.95 ± 4.07	30.30 ± 4.91	0.790
WHR	0.86 ± 0.07	0.87 ± 0.05	0.627
Physical activity and energy intake			
MET (h/day)	34.27 ± 9.32	38.0 ± 5.57	0.101
Energy intake (kcal)	2111.23 ± 694.58	2082.30 ± 707.19	0.890
Biochemistry tests			
ALT (IU/L)	18.62 ± 9.89	13.52 ± 6.62	0.043
AST (IU/L)	24.48 ± 6.65	22.32 ± 7.22	0.302
GGT (IU/L)	32.71 ± 28.92	22.80 ± 19.50	0.174
FGF-21 (pg/ml)	262.52 ± 63.77	266.05 ± 81.51	0.873
Fetuin A (mg/L)	805.02 ± 171.03	924.65 ± 227.28	0.053
Hepatic histological features			
Fibrosis grade			0.589
Grade 1 (mild)	16 (76.20)	19 (76.0)	
Grade 2 (moderate)	4 (19.0)	3 (12)	
Grade 3 (Severe)	1 (4.80)	3 (12)	
Steatosis grade			0.845
Grade 0 (normal)	6 (24.0)	–	
Grade 1 (mild)	4 (16.0)	–	
Grade 2 (moderate)	7 (28.0)	10 (40.0)	
Grade 3 (severe)	8 (32.0)	15 (60.0)	
CAP score	314.43 ± 39.58	304.0 ± 49.81	0.442

^a^Data are reported as mean ± SD for all quantitative parameters. Gender, menopause status and grade of NAFLD are presented as number (percentage).

^b^Attained from independent sample t-test except for gender, menopause status, fibrosis and steatosis grade which are obtained from Pearson chi-squared test.

*WC* waist circumference, *HC* hip circumference, *BMI* body mass index, *WHR* waist to hip ratio, *MET* metabolic equivalent, *ALT* alanine amino transferase, *AST* aspartate amino transferase, *GGT* gamma glutamyl transferase, *FGF-21* fibroblast growth factor-21, *NAFLD* non-alcoholic fatty liver disease, *CAP* controlled attenuation parameter.

**Table 2:** Physical activity, energy and several other food components intakes and their differences in the placebo and soy isoflavone groups at the baseline and at the end of the study.

Variables	Groups	Baseline	After 12 weeks	Changes (%)	P value^a^
Physical activity (MET.h.d)	Soy isoflavone	38.0 ± 5.57	39.94 ± 9.95	1.94 ± 9.75	0.329
	Placebo	34.27 ± 9.32	36.77 ± 6.76	2.50 ± 8.21	0.179
	P value^b^	0.101	0.223	**0.836**	
Total energy intake (kcal/day)	Soy isoflavone	2082.30 ± 707.19	1981.44 ± 538.58	− 100.85 ± 783.37	0.526
	Placebo	2111.23 ± 694.58	1984.72 ± 1108.32	− 126.50 ± 1235.43	0.644
	P value^b^	0.890	0.990	0.932	
Total carbohydrates (g/day)	Soy isoflavone	233.30 ± 106.29	240.71 ± 88.30	7.40 ± 129.36	0.777
	Placebo	261.72 ± 74.39	246.50 ± 146.78	− 15.22 ± 138.36	0.620
	P value^b^	0.308	0.870	0.570	
Total protein (g/day)	Soy isoflavone	74.71 ± 25.64	73.64 ± 18.62	− 1.07 ± 25.04	0.832
	Placebo	87.38 ± 41.95	90.72 ± 48.50	3.34 ± 59.31	0.799
	P value^b^	0.215	0.140	0.753	
Total fat (g/day)	Soy isoflavone	99.30 ± 34.82	85.46 ± 19.71	− 13.83 ± 36.09	0.067
	Placebo	107.05 ± 60.10	93.46 ± 30.12	− 13.59 ± 68.62	0.375
	P value^b^	0.588	0.285	0.988	
Cholesterol (mg/day)	Soy isoflavone	286.46 ± 182.51	219.94 ± 71.11	− 66.51 ± 175.48	0.070
	Placebo	238.18 ± 142.47	216.06 ± 184.52	− 22.11 ± 177.29	0.574
	P value^b^	0.330	0.928	0.40	
Fiber (g/day)	Soy isoflavone	27.61 ± 18.48	33.26 ± 22.55	5.65 ± 25.18	0.273
	Placebo	27.43 ± 11.04	30.36 ± 25.12	2.93 ± 24.13	0.584
	P value^b^	0.968	0.683	0.712	
SFA (g/day)	Soy isoflavone	21.08 ± 6.60	20.10 ± 6.04	− 0.98 ± 7.54	0.522
	Placebo	21.88 ± 6.38	20.94 ± 7.30	− 0.94 ± 7.39	0.566
	P value^b^	0.681	0.672	0.985	
MUFA (g/day)	Soy isoflavone	38.97 ± 14.16	33.07 ± 7.58	− 5.89 ± 14.41	0.052
	Placebo	37.03 ± 10.52	34.84 ± 11.40	− 2.19 ± 14.85	0.506
	P value^b^	0.608	0.534	0.397	
PUFA w-3 (g/day)	Soy isoflavone	0.85 ± 0.48	0.69 ± 0.29	− 0.15 ± 0.53	0.152
	Placebo	0.66 ± 0.47	0.66 ± 0.47	0.002 ± 0.59	0.986
	P value^b^	0.182	0.802	0.338	
PUFA w-6 (g/day)	Soy isoflavone	10.57 ± 4.88	8.53 ± 2.54	− 2.03 ± 4.34	0.028
	Placebo	9.56 ± 4.01	8.31 ± 3.91	− 1.25 ± 6.02	0.350
	P value^b^	0.457	0.817	0.614	
Vitamin C (mg/day)	Soy isoflavone	100.05 ± 99.49	102.59 ± 50.78	2.53 ± 101.45	0.901
	Placebo	122.94 ± 69.98	129.79 ± 71.87	6.85 ± 82.41	0.707
	P value^b^	0.381	0.141	0.877	
Vitamin E (mg/day)	Soy isoflavone	37.73 ± 16.76	30.68 ± 8.56	− 7.05 ± 15.14	0.029
	Placebo	35.82 ± 11.40	31.92 ± 10.21	− 3.90 ± 15.39	0.259
	P value^b^	0.660	0.657	0.489	
Selenium (mg/day)	Soy isoflavone	103.73 ± 58.27	102.90 ± 38.64	− 0.82 ± 55.52	0.941
	Placebo	110.61 ± 48.68	113.68 ± 95.39	0.57 ± 24.80	0.904
	P value^b^	0.607	0.669	0.882	
Zinc (mg/day)	Soy isoflavone	10.80 ± 3.90	11.23 ± 3.37	0.42 ± 4.15	0.617
	Placebo	16.20 ± 19.84	16.77 ± 14.50	0.57 ± 24.80	0.917
	P value^b^	0.190	0.101	0.978	

All the variables demonstrated as mean ± SD.

Significant values are in [bold].

*SFA* saturated fatty acids, *MUFA* mono unsaturated fatty acids, *PUFA* poly unsaturated fatty acids.

^a^*P* value attained from paired sample t-test.

^b^*P* value based on independent sample t-test.

### Primary outcome

As Table 3 indicates, the decrease in grade steatosis at the end of week 12 compared to before treatment was significant only in soy isoflavone group (P = 0.001). A significant change is observed between the two groups regarding the steatosis grade, before and after the trial (P = 0.005). A significant decline was also observed regarding CAP score only in the soy isoflavone group at the end of week 12 compared to baseline (304.0 ± 49.8 vs 272.20 ± 42.55; P = 0.011) (Table 4). However, no significant change in the fibrosis grade was found within and between the two groups (P > 0.05).

**Table 3:** Fibrosis and steatosis grade at the baseline and at week 12 by placebo and treatment groups.

Variables	Soy isoflavone group		P value^a^	Placebo group		P value^a^	P value^b^
	Baseline	After 12 weeks		Baseline	After 12 weeks		
Fibrosis grade							
Grade1	19 (76.0)	21 (84.0)	0.143	16 (76.20)	16 (76.20)	1.000	0.523
Grade 2	3 (12.0)	3 (12.0)		4 (19.0)	4 (19.0)		
Grade 3	3 (12.0)	1 (4.0)		1 (4.80)	1 (4.80)		
Steatosis grade							
Grade 0	0	6 (24.0)	0.001	0	1 (4.80)	1.000	0.005
Grade 1	0	4 (16.0)		0	1 (4.80)		
Grade 2	10 (40.0)	7 (28.0)		9 (42.90)	4 (19.0)		
Grade 3	15 (60.0)	8 (32.0)		12 (57.10)	15 (71.40)		

Data are presented as number (percentage).

^a^P value obtained from Wilcoxon test.

^b^P value obtained from Mann–Whitney test.

**Table 4:** Anthropometric and liver histologic variables and their changes in the placebo and soy isoflavone groups at baseline and at the end of the study.

Parameters	Groups	Baseline	After 12 weeks	Changes (%)	P value ^a^
Anthropometric indices					
Weight (kg)	Soy isoflavone	84.0 ± 18.09	82.32 ± 17.23	− 1.68 ± 5.91	0.166
	Placebo	85.42 ± 14.34	82.66 ± 11.32	− 2.76 ± 6.26	0.057
	P value^b^	0.772	0.938	**0.551**	
	P value^c^			0.354	
WC (cm)	Soy isoflavone	98.56 ± 11.99	94.86 ± 10.71	− 3.69 ± 7.30	0.018
	Placebo	97.19 ± 8.54	94.42 ± 7.09	− 2.77 ± 4.20	0.007
	P value^b^	0.664	0.871	0.612	
	P value^c^			0.888	
HC (cm)	Soy isoflavone	112.88 ± 9.74	109.09 ± 8.96	− 3.78 ± 5.14	0.001
	Placebo	113.43 ± 10.06	109.55 ± 6.29	− 3.87 ± 9.32	0.071
	P value^b^	0.852	0.844	0.968	
	P value^c^			0.870	
BMI (kg/m^2^)	Soy isoflavone	30.30 ± 4.91	28.38 ± 5.44	− 1.2 ± 4.93	0.063
	Placebo	29.95 ± 4.07	29.05 ± 3.13	− 0.89 ± 2.86	0.167
	P value^b^	0.790	0.617	0.403	
	P value^c^			0.438	
WHR	Soy isoflavone	0.87 ± 0.05	0.86 ± 0.05	− 0.003 ± 0.04	0.723
	Placebo	0.861 ± 0.07	0.863 ± 0.07	0.002 ± 0.06	0.862
	P value^b^	0.627	0.780	0.728	
	P value^c^			0.986	
Liver enzymes and CAP score					
ALT (IU/L)	Soy isoflavone	13.52 ± 6.62	9.28 ± 3.89	− 4.24 ± 6.23	0.002
	Placebo	18.62 ± 9.89	17.70 ± 9.81	− 0.92 ± 13.29	0.754
	P value^b^	0.043	0.001	0.271	
	P value^c^			0.002	
AST (IU/L)	Soy isoflavone	22.32 ± 7.22	17.42 ± 3.57	− 4.89 ± 6.70	0.001
	Placebo	24.48 ± 6.65	23.35 ± 6.34	− 1.12 ± 7.68	0.511
	P value^b^	0.302	0.001	0.082	
	P value^c^			0.001	
GGT (IU/L)	Soy isoflavone	22.80 ± 19.50	18.57 ± 8.33	− 4.23 ± 13.33	0.126
	Placebo	32.71 ± 28.92	23.63 ± 14.42	− 9.08 ± 16.03	0.017
	P value^b^	0.174	0.144	0.269	
	P value^c^			0.752	
CAP score	Soy isoflavone	304.0 ± 49.81	272.20 ± 42.55	− 31.80 ± 57.54	0.011
	Placebo	314.43 ± 39.58	316.78 ± 40.82	2.35 ± 44.69	0.812
	P value^b^	0.442	0.001	0.032	
	P value^c^			0.002	
Liver hepatokines					
FGF-21 (pg/ml)	Soy isoflavone	266.05 ± 81.51	285.14 ± 79.89	19.09 ± 79.99	0.244
	Placebo	262.52 ± 63.77	276.70 ± 67.56	14.18 ± 41.19	0.130
	P value^b^	0.873	0.704	0.801	
	P value^c^			0.220	
Fetuin A (mg/L)	Soy isoflavone	924.65 ± 227.28	1048.91 ± 241.78	124.25 ± 288.39	0.041
	Placebo	805.02 ± 171.03	887.50 ± 166.89	82.48 ± 79.21	≤ 0.001
	P value^b^	0.053	0.013	0.493	
	P value^c^			0.018	

All data are depicted as mean ± SD.

*WC* waist circumference, *HC* hip circumference, *BMI* body mass index, *WHR* waist to hip ratio, *ALT* alanin amino transferase, *AST* aspartate amino transferase, *GGT* gamma glutamyl tranferase, *FGF-21* fibroblast growth factor-21, *CAP* controlled attenuation parameter.

^a^*P* value paired sample t-test.

^b^*P* value independent sample t-test.

^c^*P* value as an indication to investigate the comparison of the mean changes related to each parameter between the two groups before and after supplementation to generate ANCOVA’s model, adjusted for the primary variable, changes in energy intake, BMI, WHR and MET.

### Secondary outcome

According to Table 4, WC (98.56 ± 11.99 vs 94.86 ± 10.71, P = 0.018 in soy isoflavone group; 97.19 ± 8.54 vs 94.42 ± 7.09, P = 0.007 in placebo group) in both groups and HC (112.88 ± 9.74 vs 109.09 ± 8.96, P = 0.001) only in the soy isoflavone group significantly decreased at the end of week 12 compared to pre-treatment. Other anthropometric parameters remained unchanged. The serum levels of ALT and AST were significantly decreased only in the soy isoflavone group at the end of the study in comparison with the study initiation: ALT (13.52 ± 6.62 vs 9.28 ± 3.89; P = 0.002) and AST (22.32 ± 7.22 vs 17.42 ± 3.57; P = 0.001). At the end of the study, the serum level of GGT decreased in both groups but this decrement was significant only in the placebo group (P = 0.017). A significant increase in the serum level of fetuin A was shown in both groups at the end of the trial, but this increase was significantly higher in the soy isoflavone group compared to the placebo group (924.65 ± 227.28 vs 1048.91 ± 241.78 in soy isoflavone group and 805.02 ± 171.03 vs 887.50 ± 166.89 in placebo group; P < 0.05). However, the changes in serum level of FGF-21 were not significant in both groups before and after the trial.

## Discussion

Based on our knowledge, this is the first randomized, double-blind, placebo-controlled, clinical trial investigating the effects of soy isoflavones on the serum levels of some hepatokines such as FGF-21 and fetuin A, as well as liver histological changes using Fibroscan in patients with NAFLD. Our findings indicate that supplementation with 100 mg/day soy isoflavones along with lifestyle modification have favorable effects in the treatment of NAFLD. Although soy isoflavone consumption showed promising effects on reducing CAP score and steatosis grade, it failed to significantly improve fibrosis grade. Also, the serum level of ALT and AST improved significantly, which confirms the previous animal research on the improving effects of soy isoflavones^16,42–44^. Also, several human studies have ended up with the same result of us using soy products or isolated soy isoflavones. According to a randomized clinical trial (RCT), a reduction in the serum level of ALT and AST was observed in low-calorie, low-carbohydrate, soy-containing group in NAFLD patients through 8 weeks^45^. In a clinical trial study, patients with NAFLD showed reduction in serum ALT and AST levels after meal replacement therapy with a commercial soy yogurt honey product for 24 weeks^46^. However, Amanat et al.^47^ have indicated that genistein supplementation has no effect on ALT and AST in patients with NAFLD. Barsalani et al. also reported that isoflavones had no effect on serum level of ALT and AST^48^. In the present study, no significant changes were observed with respect to the effect of soy isoflavone on the GGT level. In line with our findings, Maleki et al. reported no alteration in the serum level of GGT in the soy milk group of NAFLD patients after 8 weeks^49^. Also, in an experimental study, 6 weeks of genistein injection in non-alcoholic steatohepatitis rat models did not change the serum level of GGT^43^. Nonetheless, some studies have reported the effect of soy isoflavones on the reduction of serum level of GGT^16,48^. These inconsistent findings may be due to differences regarding the basic value of the serum level of liver enzymes, supplementation period, and the isoflavone structure as food ingredients or isolated forms. Studies by Mujica et al.^50^ and Soleimani et al.^51^ have reported that the interventions may not be effective in subjects with concentration of liver enzymes in normal ranges.

In the present study, soy isoflavone supplementation resulted in a significant decline in CAP score and steatosis grade. Consistent with our findings, in an animal study, rats fed with 10 or 20 mg/day of soy isoflavones showed a significant improvement in liver steatosis and significant reduction in the progression of NAFLD^14^. According to an in vivo study, administration of soy isoflavones in postmenopausal obese animal models demonstrated a significant alleviation in hepatic steatosis^52^. Another experimental study indicated a dose-dependent protective effect of soy isoflavones against hepatic steatosis in Zucker rats^53^. A RCT by Barsalani et al. clarified that 6 months of supplementation with soy isoflavones in postmenopausal women has yielded a decreased risk of hepatic steatosis progression^48^. The main underlying mechanisms by which soy isoflavone is capable of alleviating hepatic steatosis are decreasing the level of sterol regulatory element binding protein-1c (SREBP-1c) and the rate of lipogenesis by fatty acid synthase (FAS) and inducing the expression of peroxisome proliferator activated receptor (PPAR) α to stimulate the oxidation of fatty acids^14^. However, the results of the present research have shown no changes in hepatic fibrosis attributed to soy isoflavone intake. Nevertheless, several former studies reported the inhibitory effect of soy isoflavones on liver fibrosis^54–57^. The category of steatosis in terms of severity, hepatocellular ballooning and inflammation are accounted for the calculation of NAFLD score. These are the main characteristics of the disease^58^. When the severity of these features is declined, the disease improvement occurred; although sometimes the improvement in the mentioned characteristics, particularly, steatohepatitis, happens concurrent with advancement of fibrosis to cirrhosis^59,60^. In accordance with this finding an experimental study has reported that liver fibrosis caused by inflammation could be controlled independently of liver steatosis under certain circumstances^61^. Another animal study in rats fed a methionine-choline-deficit diet implied the alleviation of liver steatosis but elevation of lipid peroxidation, inflammation and liver fibrosis following treatment with diacylglycerol O acyltransferase 2 antisense oligonucleotide^62^. Thus, alleviation of ballooning and no deterioration of fibrosis are now considered as precondition of a reduction in NAFLD score^63^. In this study, the liver steatosis alleviated in response to soy isoflavone supplementation, clinically and statistically but the hepatic fibrosis remained unchanged. The significant increase level of fetuin A in both groups as a result of the present study, could be partly explained by the null effect of soy isoflavone on haptic fibrosis. However, the majority of the previous studies have reported that alleviation in NAFLD and metabolic syndrome markers is associated with decreased level of fetuin A^64–66^. Moreover, a significant positive link between BMI and fetuin-A has been reported in previous studies^67,68^. Three recent studies showed that aerobic exercise without weight change has no effect on the level of serum fetuin A^69–71^. Since in the present study, participants’ BMI has been unaffected by soy isoflavone supplementation, thus, an increasing trend in fetuin A level could be described in this way. In agreement with our finding, an interesting research article including older men has reported an increased level of fetuin A after 6 months of aerobic exercise training and weight loss. Change in VO2max is mentioned as the main cause of fetuin A elevation^72^. In the present study, the subjects of both groups were advised to increase their physical activity and follow the nutritional recommendations, although the increase in physical activity and the decrease in body weight and BMI were insignificant, but it could be the reason for the increase in the serum level of fetuin A. Based on another study, after 24 weeks of intervention with meal replacement of soy yogurt and honey products, no changes were observed with respect to fetuin A level^46^. The reason for these disparities is not clear, but it seems that disease status (i.e., NAFLD vs. obesity), drug consumption, and exercise with higher intensity (e.g., 85% of maximum heart rate) are crucial factors in determining circulating fetuin A^69–71^. This research demonstrated no changes in the serum level of FGF-21 attributed to soy isoflavone intake. Although the number of studies that have investigated the effect of soy products/isoflavones on the level of FGF-21 is limited, several studies have depicted a decreasing effect of soy products/isoflavones on the serum level of FGF-21^73,74^, while another research indicated their increasing effects^75^. Supplementation with isolated isoflavone or its consumption along with other food ingredients, the duration of soy isoflavone intake, and patients’ health status may be the main reasons for these different findings.

Applying Fibroscan which is superior to other NAFLD detective devices like sonography in regard to accuracy, enrolling subjects recently diagnosed with NAFLD prior to prescribing with strict therapies, using the varied types of soy isoflavones rather than one type, and measuring the level of FGF-21 and fetuin A as new hepatokines to predict the advancement of NAFLD are some strengths related to the present study. On the other hand, failure to measure the serum level of soy isoflavones and the small sample size are considered as the limitations which, are needed to turn over in our mind.

## Conclusion

We conclude that 100 mg/day soy isoflavone intake resulted in lowering the level of ALT, AST, CAP score and steatosis grade and increasing the level of fetuin A. Moreover, soy isoflavone consumption led to a decrease in WC and HC. However, no significant changes were observed with respect to fibrosis grade and the level of serum GGT and FGF-21.

## Data Availability

Raw data that support the findings of this study are available from the corresponding author, upon reasonable request.
